# Potential Adjunctive Role of Osteopathic Manipulative Medicine in the Management of Cancer-Related Bone Pain: A Narrative Review

**DOI:** 10.7759/cureus.106984

**Published:** 2026-04-13

**Authors:** Ambrose Loc T Ngo, Niki Gharavi Alkhansari, Chi Pham, Hong Nguyen, Monica Rubi, David Tanner

**Affiliations:** 1 College of Osteopathic Medicine, Kansas City University, Joplin, USA; 2 Internal Medicine, Kansas City University, Joplin, USA; 3 Orthopedic Surgery, HCA Midwest Healthcare, Kansas City, USA

**Keywords:** manipulative treatment, non-pharmacologic therapy, osteopathic, osteopathic manipulative medicine, pain management, palliative care

## Abstract

Osteopathic manipulative medicine (OMM) is known for its therapeutic potential on the musculoskeletal system, and its emerging role and potential benefits in oncology care are gaining attention. Patients with primary and metastatic bone cancer tend to experience pain, restricted movement, and lower quality of life due to the pathology and its treatment. This narrative review examines the mechanistic rationale and available clinical evidence supporting the use of OMM in the management of pain and functional impairment among patients with bone malignancies. A literature search was conducted using PubMed and Google Scholar to identify peer-reviewed studies published between 2005 and 2025 addressing OMM interventions in oncologic and bone cancer populations. Relevant clinical and mechanistic studies were synthesized to evaluate potential therapeutic roles and safety considerations. Limited clinical studies suggest that select OMM techniques, including myofascial release (MFR), gentle soft tissue methods, and lymphatic approaches, may contribute to improvements in pain perception, mobility, and fatigue in oncology populations. Evidence specific to primary bone malignancies remains sparse, with most data derived from small trials, observational studies, and extrapolation from broader cancer cohorts. OMM may offer a non-pharmacologic adjunct targeting biomechanical dysfunction, autonomic regulation, and lymphatic flow, which are implicated in cancer-related pain and functional decline. However, heterogeneity in study design, small sample sizes, and limited bone-specific data constrain definitive conclusions. Current evidence suggests that OMM has potential as a supportive modality in bone cancer care, particularly for symptom management and functional rehabilitation. Rigorous, well-designed clinical trials are needed to establish safety parameters, standardized protocols, and efficacy within this population.

## Introduction and background

Osteopathic manipulative medicine (OMM) is a core component of osteopathic medicine that emphasizes the interrelationship between structure and function [1,2]. While OMM is primarily used in the management of musculoskeletal disorders, there is increasing interest in its potential applications in other clinical settings, including oncology [1]. OMM utilizes hands-on techniques to address somatic dysfunction and support physiologic processes such as circulation, lymphatic flow, and autonomic regulation [1-3]. Patients with primary or metastatic bone cancer frequently experience significant pain, reduced mobility, and impaired quality of life [4,5]. Conventional treatments, including surgery, chemotherapy, and radiotherapy, primarily target disease control but may not fully address symptom burden or functional limitations [6,7]. As a result, there is growing interest in integrative approaches that may complement standard oncologic care [6-8].

Emerging literature suggests that OMM may offer supportive benefits in oncology populations, including potential improvements in pain, mobility, and overall well-being [8,9]. Proposed mechanisms include modulation of musculoskeletal dysfunction, enhancement of lymphatic and circulatory dynamics, and influence on autonomic nervous system activity [10-13]. However, the current evidence remains limited and heterogeneous, particularly in patients with bone malignancies.

Therefore, the aim of this narrative review is to synthesize the existing literature on the role of OMM in patients with primary and metastatic bone cancer, with a focus on pain management, functional outcomes, and its potential integration into multidisciplinary oncology care.

## Review

Methods

This study represents a narrative review conducted using a structured literature search to evaluate the potential role of OMM in patients with primary and metastatic bone malignancies. Because this work is a narrative review, study selection was performed to guide thematic synthesis rather than to conduct a formal systematic review or meta-analysis.

A comprehensive search of PubMed and Google Scholar was performed to identify relevant peer-reviewed articles published between 2005 and 2025. Google Scholar was included to ensure broader capture of relevant literature, including studies not indexed in traditional biomedical databases. Searches were limited to English-language, peer-reviewed publications within the specified date range. The search strategy incorporated Boolean operators (AND, OR) and included terms such as “osteopathic manipulative medicine and malignancies,” “osteopathic medicine with cancer,” “osteopathic treatment effects,” “osteopathic treatment and bone cancer,” and “bone cancer treatment and care.” For Google Scholar, the first several pages of results were screened to identify the most relevant studies. Reference lists of included articles were also manually reviewed to identify additional pertinent studies.

Studies were included if they were published in English between 2005 and 2025, involved human subjects, and evaluated OMM or osteopathic manipulative treatment (OMT) in oncology populations, with particular relevance to bone cancer or cancer-related symptoms such as pain, mobility, or quality of life. Studies were excluded if they did not involve OMM/OMT as a primary intervention, focused solely on non-osteopathic therapies (e.g., chiropractic or standard physical therapy), lacked relevant clinical outcomes, were non-human studies, or were editorials, opinion pieces, or non-peer-reviewed publications.

In total, 3,032 records were identified through database searching. After removal of duplicates, 2,260 records remained for title and abstract screening. Following this screening process, 165 articles were selected for full-text review. Of these, 123 articles were excluded due to inability to access the full text, lack of relevance to OMM interventions, insufficient outcome data, or failure to meet the scope of this narrative review. Ultimately, 42 references were included in the final descriptive synthesis.

Data extracted from selected studies included study design, population characteristics, intervention type, reported outcomes, and safety considerations. Findings were synthesized descriptively and organized into thematic sections addressing pathophysiology, pain mechanisms, lymphatic considerations, palliative applications, and safety considerations.

Main findings

Pathophysiology of Bone Cancer and Malignancy

Bone cancer includes primary malignancies such as osteosarcoma, chondrosarcoma, Ewing sarcoma, fibrosarcoma, and undifferentiated pleomorphic sarcoma [14-16]. Osteosarcoma, common in adolescents, arises from osteoblasts, while chondrosarcoma primarily affects adults over 40 [17]. Ewing sarcoma originates from bone or soft tissues and is most prevalent in children and young adults [18]. Bone metastases occur when cancer spreads from primary tumors in the breast, lung, prostate, or kidney [19]. Symptoms include persistent pain, fractures, and systemic energy depletion, with patients also experiencing anxiety and depression, exacerbating their physical limitations [20-22].

As an adjunctive therapy, OMM complements conventional cancer treatments by addressing musculoskeletal, circulatory, and lymphatic dysfunctions, potentially reducing pain and improving mobility [23]. Techniques such as lymphatic mobilization may aid cancer patients with lymphedema and enhance recovery from intensive treatments. By relieving pain, increasing mobility, and reducing fatigue, OMM may help improve symptom management. Soft tissue techniques ease muscle spasms, while high-velocity, low-amplitude (HVLA) adjustments restore joint function [24]. Myofascial release (MFR) decreases inflammation, and craniosacral therapy promotes parasympathetic activity, reducing stress-induced fatigue [25]. Lymphatic pump techniques improve drainage, aiding waste removal and fluid balance [26].

OMM in Pain Management for Bone Cancer Patients

Bone cancer pain involves both nociceptive and neuropathic components due to tumor-induced osteolysis, inflammation, and nerve involvement. Cancer cells stimulate osteoclasts via receptor activator of nuclear factor kappa-Β ligand (RANKL), leading to bone resorption, microfractures, and activation of periosteal pain receptors [27]. Pro-inflammatory cytokines like interleukin-1 (IL-1), interleukin-6 (IL-6), and tumor necrosis factor-alpha (TNF-α) sensitize nociceptors, while prostaglandin E2 (PGE2) amplifies pain and inflammation [27,28]. Tumor growth can compress or infiltrate nearby nerves, causing sharp, radiating neuropathic pain and chronic pain syndromes. Additionally, periosteal nerve stimulation contributes to pain as tumors stretch and disrupt the periosteum, the bone's outer layer [28,29]. The proposed intersections between bone cancer pain mechanisms and potential osteopathic interventions are summarized in Table 1. These mechanisms provide a conceptual framework for understanding how OMM may interact with cancer-related pain pathways.

**Table 1:** Proposed mechanisms of OMM in bone cancer pain management Modulation of autonomic tone, reduction of somatic dysfunction, improved circulation and lymphatic flow, activation of central pain modulation, including descending inhibitory pathways, and potential symptom relief OMM: osteopathic manipulative medicine; TNF-α: tumor necrosis factor-alpha; RANKL: receptor activator of nuclear factor kappa-Β ligand; HVLA: high-velocity, low-amplitude

Category	Key Concepts	Representative References
Bone Cancer Pain Mechanisms	Osteolysis, RANKL-mediated osteoclast activation, periosteal nerve stimulation, inflammatory cytokines (IL-1, IL-6, TNF-α), neuropathic compression	Halvorson 2006 [27]; Mantyh 2014 [28]
OMM Techniques Potentially Applicable	Gentle soft tissue techniques, myofascial release, indirect techniques, selected cranial techniques (avoiding HVLA in fragile bone)	Gustowski 2024 [23]
Proposed Physiologic Effects	Modulation of autonomic tone, reduction of somatic dysfunction, improved circulation and lymphatic flow, activation of central pain modulation, including descending inhibitory pathways, and potential symptom relief	Muñoz-Gómez 2022 [25]; Remien 2025 [29]

Given the multifaceted nature of chronic pain, a comprehensive management approach is essential. The five models of osteopathic medicine, including biomechanical, respiratory-circulatory, neurologic, metabolic-energy, and behavioral, provide a framework for incorporating OMT into pain management [23]. Among the most studied OMT techniques are soft tissue therapy (STT), MFR, and osteopathic cranial manipulative medicine (OCMM).

OCMM focuses on the skull, sacrum, and their interconnected membranes, and has been associated with improvements in headaches, migraines, and stress-related pain [25]. By regulating the autonomic nervous system, OCMM balances sympathetic and parasympathetic activity, alleviating tension headaches and improving nerve function [25]. Techniques such as balanced membranous tension, V-spread, and compression of the fourth ventricle are commonly used to reduce craniosacral restrictions and enhance pain relief.

OMM in Cancer Pain Management: A Multimodal Approach

Cancer pain care involves a combination of pharmacological and non-pharmacological treatments, tailored to address the multifaceted nature of cancer pain. Non-pharmacological treatments complement pharmacological efforts, with radiation therapy targeting painful bone metastases and surgery aimed at stabilizing bones and preventing fractures. Palliative care provides a holistic approach to managing pain, focusing on improving quality of life. Additionally, OMM techniques offer non-invasive, individualized, and comprehensive pain relief by addressing multiple pain pathways, including musculoskeletal ones, while reducing the reliance on medications or surgical interventions [30]. However, there can be potential challenges and limitations to consider when implementing OMM in cancer care.

OMM and Quality of Life in Malignancy Patients

While OMT is generally avoided in patients with bone pathologies, including osteoporosis and local malignancy, due to safety concerns [31], some studies suggest its benefits in improving pain perception, functionality, and mobility in cancer patients. A nonrandomized controlled trial on 24 geriatric oncology patients receiving either OMT or physical therapy for four weeks post-surgery assessed pain and quality of life using the Numeric Rating Scale (NRS) and Quality of Life Questionnaire Core 30 (QLQC30) [32]. OMT techniques such as soft tissue, rib raising, MFR, and suboccipital release were well tolerated and did not impose high physical demands. The experimental group showed significant pain reduction at the two- and four-week marks, an effect not observed in the control group. While studies specifically on OMT and bone cancer mobility are scarce, a case report on a type 1 chondrosarcoma patient incorporated manual therapy to improve hip and knee range of motion and reduce postoperative edema [33,34].

In pediatric oncology, a study on 23 patients receiving weekly OMT, including MFR, muscle energy, balanced ligamentous tension, and visceral manipulation, assessed constipation and pain using the Bristol Stool Scale and FACES Scale [35]. Among participants, results showed no worsening of symptoms. Patients reported regular bowel movements and stable or lower pain scores, indicating that OMT appears to be well tolerated in this population, although its efficacy in improving function and pain remains to be further established.

OMT has also been linked to improvements in emotional well-being and fatigue in cancer patients, though studies in bone cancer populations are limited. A 2018 study by Steel et al. examined patient-reported outcomes on OMT perception, fatigue, pain, and insomnia in a palliative care setting [36]. Patients diagnosed with various cancers, including breast, lung, hematologic, prostate/urologic, head and neck, and melanoma, found OMT calming, with reported reductions in pain, insomnia, and edema. A randomized controlled trial found that a 12-day physiotherapy program, including active exercises, MFR, and proprioceptive neuromuscular facilitation, significantly reduced fatigue, drowsiness, pain, and lack of appetite, improving patients’ general well-being [37].

However, careful technique selection is essential to prevent harm, given the compromised skeletal integrity from malignancy [38]. Key safety considerations and contraindications are summarized in Table 2. Soft tissue techniques, MFR, and viscerosomatic methods have been identified as safe and effective for improving quality of life in cancer patients, including those with bone pathologies [32,33,35]. Lymphedema, caused by lymphatic stasis, can negatively impact post-treatment recovery in bone cancer patients [39].

**Table 2:** Potential risks and contraindications of OMM OMM: osteopathic manipulative medicine

Challenges and Limitations of OMM in Cancer Care	References
Severe bone fragility: Patients with advanced metastatic bone disease or osteoporosis are at increased risk of fractures. High-velocity manipulative techniques are contraindicated, and gentler approaches should be considered.	Cancer Research UK, 2022 [40]
Metastatic spread: Concerns exist that certain OMM techniques, particularly those involving lymphatic treatments, could facilitate cancer spread. While debated, caution is advised when treating patients with active metastases.	Remien et al., 2024 [29]
Coagulopathies: Patients with low platelet counts or bleeding disorders may face complications from even gentle manipulation. Evaluating coagulation status is crucial before administering OMM.	Cancer Research UK, 2022 [40]

Osteopathic Manipulative Medicine and Palliative Care in End-Stage Cancer

End-stage cancer patients often experience severe pain, restricted mobility, and psychological distress. While conventional palliative care relies heavily on pharmaceuticals, OMM represents a potential complementary, non-invasive approach. Techniques like MFR and cranial-sacral therapy have been reported to ease musculoskeletal pain and promote relaxation [41]. A clinical trial in Milan, Italy, found significant pain reduction in geriatric oncology patients receiving OMT alongside physiotherapy, though quality-of-life improvements were not statistically conclusive [32]. Additionally, qualitative studies indicate that patients report reduced fatigue, improved sleep, and overall well-being after OMM [36].

OMM’s non-invasive nature makes it particularly beneficial for frail patients, as it minimizes physiological stress while effectively managing symptoms. A pilot study on pediatric oncology patients demonstrated its safety and feasibility, showing reduced pain with no adverse effects [35]. Further research suggests OMT may help alleviate constipation and shorten hospital stays in pediatric cancer patients [35]. A meta-analysis of OMT in palliative care found significant pain reduction and improved patient comfort [42]. Another study evaluating patient experiences in palliative care found that individuals receiving osteopathic treatment alongside conventional therapy described it as a holistic, meditative, and valuable non-pharmaceutical approach [36].

Discussion

Key Findings

The current evidence supporting OMM's role in cancer care is limited by a lack of large-scale randomized controlled trials. Many studies remain small or observational, limiting their generalizability. Overall, the available evidence is heterogeneous and primarily low to moderate in quality, which constrains the strength of conclusions that can be drawn. A nonrandomized clinical trial found that while OMM provided significant pain relief in geriatric oncology patients, improvements in quality of life were not statistically significant [32]. Challenges in conducting larger trials include difficulties in standardizing OMM protocols and implementing placebo controls. Additionally, the incorporation of OMM into conventional cancer treatment faces several barriers, including skepticism from healthcare providers and logistical difficulties in coordinating treatment plans between osteopathic practitioners and oncologists. Additionally, a study investigating the safety and feasibility of OMM in pediatric oncology patients found that while OMM could be safely administered, careful patient selection and technique modification were necessary to minimize risks [35]. OMM may also influence central pain processing through activation of descending inhibitory pathways, including serotonergic and noradrenergic systems, which are known to modulate nociceptive transmission at the spinal level [25,29]. However, these mechanistic findings are largely extrapolated from broader pain literature and have not been consistently validated in oncology-specific populations. This mechanism may contribute to reductions in perceived pain and improved symptom control in patients with cancer-related pain. 

Limitations

A potential limitation of our review is that we only included articles published within the past 20 years; this could have potentially limited the inclusion of earlier foundational studies. However, as the body of evidence evolves, we aimed to capture the most up-to-date evidence. Studies were also categorized by subtopic, with articles with a higher level of evidence and more recent years of publication given priority. Due to limited articles on certain subtopics, we aimed to include a minimum of three articles within each subtopic category. Additionally, the included studies demonstrate variability in study design, patient populations, and outcome measures, further limiting direct comparison and synthesis of findings. The limited number of articles identified in our review further highlights the need for more robust research on OMM use in integrative oncology care. Lastly, the findings of this review may not be generalizable to all patients with bone cancer due to the potential confounding variables of patients, such as the staging of cancer and types of bone cancer.

Future Directions

In summary, this review highlights OMM as a potential therapy for patients with primary or metastatic bone cancer experiencing symptoms, by relieving pain, restoring mobility, and enhancing the patient’s overall quality of life. However, current evidence primarily supports feasibility and potential benefit, and definitive conclusions regarding efficacy remain limited. To establish OMM's importance in oncology care, further studies are necessary, as noted in our limitations. Future research efforts should focus on larger, multicenter, randomized clinical trials that can provide more robust data, making it easier to demonstrate the effectiveness and safety of OMM in cancer treatment. We also recommend that future studies use measurable outcomes to assess the efficacy of OMM in terms of pain alleviation, restoration of functional abilities, and enhancement of life quality via patient-reported outcomes and recommend using standard reporting guidelines (such as CONSORT-PRO) to allow for study reproducibility.

## Conclusions

OMM may have potential as an adjunct treatment for cancer patients, particularly those with bone cancers. By addressing the skeletal system and muscles, enhancing lymphatic drainage, and promoting relaxation, OMM provides benefits that not only reduce pain and improve mobility but also contribute to the overall emotional and physical well-being of patients throughout their cancer journey. Integrating OMM into oncology care allows for a more comprehensive, multi-dimensional approach that complements conventional cancer treatments. Overall, OMM may serve as a valuable tool for symptom management. However, further collaboration and research are essential to establish evidence-based best practices.
